# Recent Insights on Post-COVID in Pediatrics

**DOI:** 10.1097/INF.0000000000003976

**Published:** 2023-04-20

**Authors:** Elena Chiappini, Roberta Pellegrino, Cristiana M. Nascimento-Carvalho, Luisa Galli

**Affiliations:** From the § Department of Paediatrics, Federal University of Bahia School of Medicine, Salvador, Brazil.; ‡ Postgraduate School of Pediatrics, Department of Health Sciences, University of Florence, Florence, Italy; † Infectious Diseases Unit, Meyer Children’s Hospital IRCCS; * Department of Health Sciences, University of Florence

**Keywords:** post-COVID, children, SARS-CoV-2

Shortly after the start of the COVID-19 pandemic, reports of previously healthy individuals experiencing protracted symptoms after SARS-CoV-2 infection started to arise. In May 2020, the term long-COVID was launched as a Twitter hashtag among patient support groups.^1^ Therefore, long-COVID is the first clinical condition detected by patients earlier than healthcare professionals. Since then, many authors have used different terms for the condition, including long-haulers, COVID-long, postacute sequelae of COVID-19 (PASC), post-COVID, COVID syndrome and long-COVID.^2^

The UK National Institute of Health and Care Excellence (NICE) used the word “long-COVID” to describe signs and symptoms that continue or develop after acute COVID-19, including both “ongoing symptomatic COVID-19” (from 4 to 12 weeks after onset) and “post-COVID syndrome” (12 weeks or more after onset).^3^

The World Health Organization issued a clinical definition of post-COVID in adults as a condition occurring in people with a history of probable or confirmed SARS-CoV-2 infection with persisting or new symptoms 3 months after the onset, lasting for at least 2 months and which cannot be explained by an alternative diagnosis.^3^ A research definition of post-COVID in young people was proposed in April 2022, emphasizing that it occurs in patients with a history of confirmed SARS-CoV-2 infection with one or more persisting physical symptoms for at least 12 weeks after infection; the symptoms should affect daily functioning, may continue or develop after COVID-19 and may fluctuate or relapse.^3^

## EPIDEMIOLOGY AND RISK FACTORS

The reported prevalence of post-COVID in children and adolescents is highly variable, ranging from 1.6% to 70% in different studies.^4^ This significant variability may have several explanations, such as the inclusion of a heterogeneous group of children in initial studies, which were often of poor quality and had small sample sizes.^2,5^ Notably, early studies were undermined by recall biases since most of them were based on surveys or questionnaires, and control groups were lacking.^2,4^

Adolescent age, female sex, overweight, allergic diseases and long-term health conditions have been reported to be associated with an increased risk of post-COVID.^2,4^ Among hospitalized patients, in-patient stay of >48 hours and 4 or more symptoms at admission have been associated with post-COVID.^5^ In contrast, no correlation between acute illness severity and persistence of symptoms has been identified.^4^

## CLINICAL PRESENTATION

**FIGURE 1.**
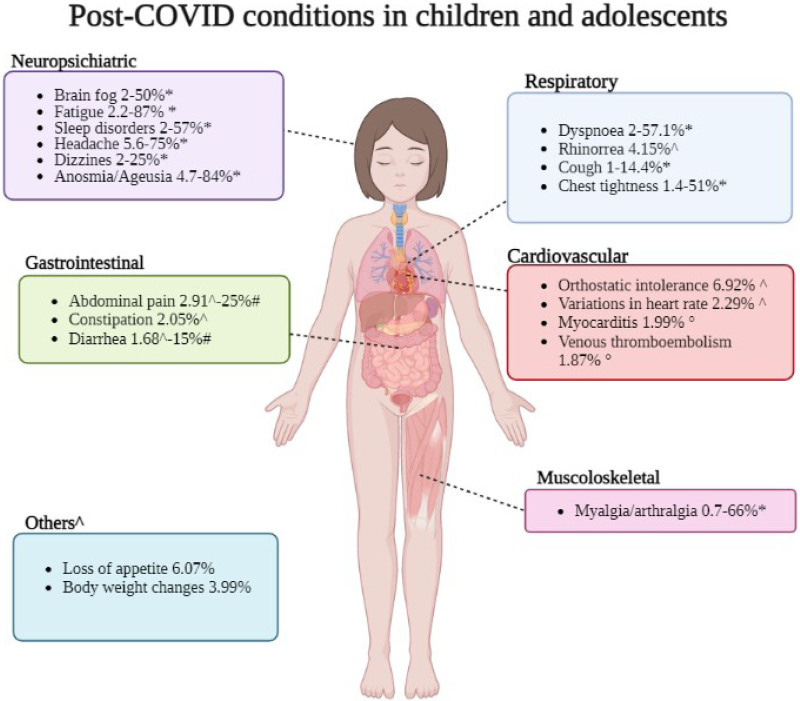
Post-COVID conditions in children and adolescents. *Pellegrino et al.^4^ ^Lopez-Leon et al.^2^ #Behnood et al.^6^ °Kompaniyets et al.^14^

The most commonly reported features post-COVID in children include fatigue (47%), dyspnea (43%), headache (35%), cognitive difficulties (26%), myalgia, (25%), abdominal pain (25%), anosmia (18%), fever (18%), cough (17%) and diarrhea (15%) (Fig. 1).^6^

Clustering of symptoms has also been noted in post-COVID conditions, such as cardiorespiratory (tachycardia, chest pain, fatigue and postexertional malaise) or clustering of gastrointestinal symptoms (recurrent abdominal pain, nausea and alteration in bowel habits), neuropsychiatric symptoms (chronic headache, brain fog, difficulty concentrating and mood disorders) or musculoskeletal symptoms (arthralgia and muscle pain).^7^ Moreover, children experiencing gastrointestinal symptoms tend to improve faster than those with fatigue, cardiorespiratory symptoms and headache.^7^

Post-COVID symptoms tend to decline over time, as confirmed by a matched-cohort longitudinal study, which included 5086 nonhospitalized children (2909 SARS-CoV-2 positive and 2177 negative).^8^ In the same study, new complaints, particularly tiredness, shortness of breath, poor well-being and fatigue, were reported for the first time at 6 and 12 months from acute infection for both cases and controls suggesting that these symptoms could be related to multiple factors in addition to SARS-CoV-2.^8^ Similarly, many of the post-COVID symptoms have been commonly reported among young people despite their SARS-CoV-2 status. In particular, a high prevalence of poor mental health and low well-being scores among children age 11–17 years was found throughout the pandemic and, when compared to a control group, no difference was noted between those with a previous history of SARS-CoV-2 infection and controls.^9^ Actually, some of the abovementioned conditions may have a functional nature, which applies primarily to symptoms like fatigue, malaise, mood changes and headache, making it difficult to ascertain whether these disorders are secondary to SARS-CoV-2 infection or are indirectly linked to lifestyle changes and stressful circumstances experienced by children and adolescents during pandemic lockdown.^2,4^

A meta-analysis focusing on controlled studies found that the frequency of the most reported persistent clinical features was similar among SARS-CoV-2-positive cases and controls, except for anosmia, headache and cognitive symptoms.^6^ Moreover, higher study quality was associated with a lower prevalence of all symptoms.^6^

In contrast, the systematic review and meta-analysis by Lopez-Leon et al. including 80,071 children and adolescents from 21 studies, reported that children infected by SARS-CoV-2 had a higher risk of persistent dyspnea (odds ratio [OR] 2.69; 95% confidence interval [CI] 2.30–3.14, I2 0%), anosmia/ageusia (OR 10.68; 95% CI 2.48, 46.03, I2 0%), and fever (OR 2.23; 95% CI 1.2–4.07, I2 12%) than controls.^2^ However, only 3 of the 21 included studies (accounting for 364 of 80,071 patients) were based on clinical assessment, and most of the data relied on phone or electronic surveys/questionnaires or health insurance data. Therefore, a possible overestimation of self-reported symptoms needs to be considered.

More recent observational studies, which included clinical follow-up and assessment, found that post-COVID prevalence ranged from 10% to 30%, with fatigue being the most frequently reported symptom (6.5% to 70%).^10–13^ In particular, a study conducted in Mexico, including 215 children with a documented SARS-CoV-2 infection found a dyspnea prevalence of 8.8% at 2 months from acute SARS-CoV-2 infection, which decreased to 2.3% at 4 months, likewise rhinorrhea and dry cough.^10^ Smell and taste alterations were rare and reported with a prevalence of 1.4%.^12^

A retrospective cohort study by the Centers for Disease Control and Prevention (CDC), based on healthcare insurance data, showed that children and adolescents with previous COVID-19 had a higher risk of acute pulmonary embolism, myocarditis, cardiomyopathy, venous thromboembolic event, renal failure and type 1 diabetes.^14^ Cardiologic alterations have been described at echocardiography and electrocardiogram in children with post-COVID symptoms, but these findings resolved over time.^4^

Among 51 children and adolescents hospitalized with COVID-19, 5 (10%) had persistent symptoms compatible with post-COVID, and 2 (4%) reported shortness of breath >13 months after hospitalisation.^15^ In a smaller sample of 16 children (median age 7.5 years) with previous asymptomatic or paucisymptomatic SARS-CoV-2 infection, no abnormalities were found on lung ultrasound, airway resistance test, forced spirometry and diffusing capacity of the lungs for carbon monoxide during a follow-up of at least 30 days. This suggests that children might be less prone to develop pulmonary complications than adults.^16^ In contrast, 54 post-COVID-19 children and adolescents (mean age 11 ± 3 years) and 9 healthy controls (mean age 10 ± 3 years) were investigated by morphologic and free-breathing phase-resolved functional low-field magnetic resonance imaging. In the COVID-19 group, 25 (46%) were diagnosed with post-COVID at enrolment, and persistent pulmonary dysfunction was found in both the post-COVID group and those who recovered from COVID-19.^17^ Further studies are needed to assess the effects of the virus on the respiratory system after mild and severe COVID-19 in children, and how these effects are different from other respiratory viral infections.

## PATHOGENESIS

Post-COVID is proposed to have a multifactorial pathogenesis, including persisting viral-induced inflammation, immune dysregulation, autoimmunity, diffuse endothelial damage and microthrombosis.^18^ SARS-CoV-2 uses angiotensin-converting enzyme 2 as a gateway to invade human cells. Prolonged viral shedding after the acute infection has been documented in the respiratory tract, feces and intestinal biopsies, which could lead to sustained inflammation and a profibrotic state.^4,7^ In adults, for example, pulmonary fibrosis has been described as part of post-COVID respiratory sequlae.^19^

Concerning immune dysregulation, T-cell alterations have been proposed as drivers of post-COVID. T-cell subsets polarize toward an exhausted/senescent state of CD4+ and CD8+ T cells and perturbances in CD4+ Tregs, potentially resulting in unresolved inflammation at 6 months from acute infection.^19^ Similarly, children with persistent symptoms after acute COVID-19 have been shown to have higher levels of interleukin-6 (IL-6) and interleukin-1β (IL-1β) which are important mediators of inflammation.^20^ This persistence of the inflammatory state could manifest as persisting fatigue and headache weeks after acute infection.^19,20^ SARS-CoV-2 infection could also potentially induce autoantibodies through molecular mimicry. Autoantibodies against G-protein coupled receptors (GPCRs), which can alter the neuronal and vascular process, have been documented after COVID-19 in adults and could explain some neurological and cardiovascular symptoms.^20^

In addition, several post-COVID symptoms may be related to the potential neurotropism of SARS-CoV-2. Virus particles have been found in the olfactory neuroepithelium and could explain smell alterations.^21^ Moreover, some authors suggested that persistent cough could result from the involvement of vagal sensory nerves. Many of the symptoms associated with post-COVID such as fatigue, sleep disorders, orthostatic intolerance, cognitive symptoms, body temperature dysregulation and altered bowel habits are commonly present in dysautonomia, which could result from the SARS-CoV-2 infection or immune-mediated effected associated with altered cytokine levels.^2^ SARS-CoV-2-related neuroinflammation is supported by radiological findings showing cerebral metabolic and perfusion defects in some children with post-COVID conditions.^4,7^

Postinfective fatigue syndrome (PIFS) has been described following other infections such as Epstein-Barr virus, dengue virus, chikungunya virus, Ebola virus and other coronaviruses such as SARS and Middle East Respiratory Syndrome virus (MERS).^6,22^ The pathophysiology of PIFS is still unclear, but it is possible that the viral illness may act as a precipitating factor alongside other concomitant environmental triggers, such as distressing factors. Therefore, postviral fatigue needs a careful clinical characterization, including standardized investigations and a broad evaluation of physical and mental health.^22^

## MANAGEMENT

Since the exact impact and consequences of post-COVID condition in children has not been fully established, its management is still a matter of debate and, therefore, pertinent consensus guidelines are lacking. Some authors do not recommend a structured follow-up for all children with COVID-19 because most symptoms are mild, nonspecific and usually self-resolving.^23^

On the other hand, if post-COVID syndrome is suspected, it might be appropriate to exclude organic disorders and optimize daily function and quality of life. The American Academy of Pediatrics has proposed a stepwise approach, stating that all children with a previous SARS-CoV-2 infection should be assessed by a primary care pediatrician between 4 and 12 weeks from onset to identify symptoms which interfere with daily functioning, as well as help enforce and aid a return to healthy lifestyle habits.^24^ Only if the symptoms persist beyond 12 weeks and/or impact the patient’s daily functioning, additional diagnostic testing should be considered. For instance, in case of persistent respiratory symptoms or exercise intolerance in children older than 6 years, an extensive cardiopulmonary evaluation is suggested. Moreover, the American Academy of Pediatrics advises to refer children and adolescents with suspected post-COVID conditions to a dedicated multidisciplinary post-COVID-19 clinic and, only if this is not available, to a pediatric subspecialist according to symptoms.^24^

In conclusion, the robustness of reported post-COVID condition in children is very limited since most of the available studies are biased by heterogeneity in definition and assessment methods; as such, the causality link between SARS-CoV-2 infection and persistence of the wide range of reported symptoms is weak.^25^ Moreover, most studies and recommendations on investigations and management of post-COVID were the result of experiences in the early pandemic period, when the perception of SARS-CoV-2-related postacute symptoms was likely to be overestimated. Hence, high-quality controlled studies in recently infected children are needed to better define this condition and its optimal management.
